# Influence of Soluble and Mineral Sources of Cobalt on Methanogenesis and Growth in *Methanosarcina barkeri* Fusaro and *Methanococcus maripaludis* S2

**DOI:** 10.1111/1462-2920.70482

**Published:** 2026-11-10

**Authors:** Hunter Dulay, Md Gahangir Alam, Devon Payne, James Larson, Brian Bothner, Eric S. Boyd

**Affiliations:** ^1^ Department of Microbiology and Cell Biology Montana State University Bozeman Montana USA; ^2^ Department of Chemistry and Biochemistry Montana State University Bozeman Montana USA

**Keywords:** cobalt sulphide, cobaltoan pyrite, cobamide, methyltransferase, mineral dissolution

## Abstract

Cobalt (Co) is a required component of cofactors and coenzymes in all life and has a pronounced role in the one carbon metabolism of methanogens. However, methanogens often inhabit sulphidic environments where Co precipitates and is incorporated into low solubility mineral phases, such as pyrite (FeS_2_). Here, we initially examined the influence of Co availability on the growth kinetics of (1) *Methanosarcina barkeri* with different methanogenesis substrates and (2) *Methanococcus maripaludis* with different sulphur sources. We then investigated Co acquisition from cobaltoan pyrite [(Co,Fe)S_2_]. Co demand was highest in *M. barkeri* grown on methanol/acetate relative to hydrogen/carbon dioxide or methanol only and in *M. maripaludis* grown with sulphide relative to cysteine or cysteine/thiosulphate. Methanol/acetate‐grown *M. barkeri* and formate‐grown *M. maripaludis* grew with (Co,Fe)S_2_ as well as with soluble sources of Co, Fe and S. Both strains required direct contact to reduce and solubilize Fe and S from (Co,Fe)S_2_. Further, *M. barkeri* grown with (Co,Fe)S_2_ produced an equal amount of cobamides relative to cells provided with soluble Co, Fe, and S. These findings indicate that the availability of methanogenesis substrates and sulphur sources influence Co requirements for methanogens and that these organisms can acquire Co, Fe and S from (Co,Fe)S_2_.

## Graphical abstract

Mode of methanogenesis and sulphur source affected cobalt (availability and biosynthetic demand in cultures of *Methanosarcina barkeri* Fusaro and *Methanococcus maripaludis* S2. Both strains reductively dissolved cobaltoan pyrite, (Co,Fe)S_2_, as a source of iron, Co and sulphur and assimilated the mobilized Co as cobalamide cofactors. These findings provide insight into factors that influence Co demands in methanogens and show that mineral sources of Co that prevail under sulphidic conditions commonly inhabited by methanogens are bioavailable.

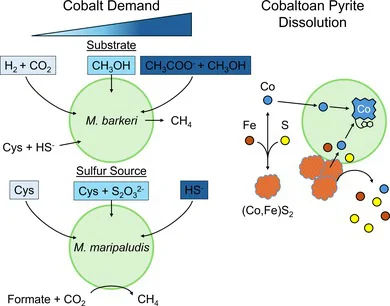

## 1 Introduction

Cobalt (Co) is utilized by all forms of life as a component of cofactors and coenzymes (Williams and da Silva 2005) and is a required trace element (Remick and Helmann 2023). The widespread utilization of Co likely stems from incorporation of this metal into the metabolism of ancestral cells early in Earth history when the metal was more widely available in its soluble form than today (David and Alm 2011; Zerkle et al. 2005). Consistent with this hypothesis, bioinformatic analysis of contemporary microbial communities (Swaney et al. 2022), as well as sequenced genomes (Shelton et al. 2019), reveals that upwards of 86% of organisms code for cobamide‐dependent enzymes. Cobamides, such as cobalamin or vitamin B_12_, are a class of Co‐containing cofactors that are involved in varied metabolic pathways (Remick and Helmann 2023) including methionine biosynthesis (Roth et al. 1996), reductive dehalogenation (Banerjee and Ragsdale 2003), bacteriochlorophyll synthesis (Gough et al. 2000), CO_2_ fixation by carbon monoxide dehydrogenase complex (CODH) and the reductive acetyl‐CoA pathway (Wood et al. 1982; Wagner et al. 2016) and methanogenesis (Thauer et al. 2008), among others. As such, the availability of Co over Earth's history has likely had a profound influence on the origin (Russell 2022) and evolution of life and the geochemical cycles that life modulates (Zerkle et al. 2005; Dupont et al. 2010; Anbar and Knoll 2002; Anbar 2008).

The average crustal abundance of Co is 25 ppm (Taylor 1964), though concentrations in ultramafic rocks can be as high as 200 ppm (Hamilton 1994; Dehaine et al. 2021). Despite a relatively high amount of Co in the crust, the amount of Co mobilized by weathering and delivered via rivers to oceans only accumulates to sub nM concentrations (Zerkle et al. 2005). This is likely due to a combination of its importance as a nutrient, resulting in its assimilation, and its limited solubility. According to the Irving‐Williams ordering of the stability of complexes of bivalent metal ions (Pd > Cu > Ni > Co > Zn > Cd > Fe > Mn > Mg; (Irving and Williams 1948)) Co(II)‐complexes are among the most stable regardless of ligand (either oxygen or sulphur). This leads to sequestration via sorption on metal oxide/sulphide surfaces or direct co‐precipitation as sulphide (HS^−^) complexes (Dehaine et al. 2021; Moore et al. 2018; Krupka and Serne 2002). Under sulphidic (euxinic) conditions Co readily reacts to form aqueous metal sulphide clusters (Rickard and Luther 2006) and ultimately sulphide minerals (Swanner et al. 2019). The reactivity of Co with HS^−^, combined with the episodic euxinic conditions in oceans from the late Archean to the late Cretaceous (Canfield 1998; Scott et al. 2008, 2011; Gill et al. 2011) and decreased input by hydrothermal systems (Swanner et al. 2014), likely drove a decrease in Co from mid nM levels to sub nM levels (Zerkle et al. 2005; Anbar 2008) due to the insolubility of cobalt sulphide (CoS; K_sp_ = 10^−10^ M; (Rickard and Luther 2006)). This raises the question as to how Co was acquired by microorganisms under euxinic conditions.

Methanogens are anaerobic archaea that generate methane (CH_4_) as a byproduct of their metabolism of carbon dioxide (CO_2_), alcohols and organic acids, among others substrates (Ferry 1994). All methanogens require high amounts of Co (Glass and Orphan 2012; Scherer et al. 1983) to synthesize cobamide cofactors that are necessary for sodium‐pumping methyltransferase complex (Mtr) and CODH. Thus Co is essential to the energy metabolism and/or assimilatory carbon metabolism of cells (Thauer et al. 2008). The requirement for Co is even greater in methylotrophic methanogens where additional Co‐dependent methyltransferase enzymes are required (Glass and Orphan 2012; Scherer et al. 1983). Yet, methanogens often inhabit euxinic habitats (Liu et al. 2012) where Co is likely sequestered as soluble metal aqueous clusters (Rickard and Luther 2006) or as low solubility metal sulphides (Swanner et al. 2019). Indeed, methanogens have continuously inhabited Earth for the past > 3.8 Gy (Ueno et al. 2006; Wolfe and Fournier 2018; Weiss et al. 2016), spanning periods of comparatively high to low Co availability (Zerkle et al. 2005; Dupont et al. 2010; Anbar and Knoll 2002; Anbar 2008). As such, methanogens represent model organisms to examine Co requirements and acquisition mechanisms under conditions that impose apparent Co limitation.

Potential insight into how methanogens obtain thiophilic metals, such as Co, in euxinic environments comes from several studies that indicate these organisms can use soluble iron sulphide aqueous clusters (FeS_aq_) and low solubility iron sulphides, such as mackinawite (FeS) and pyrite (FeS_2_), as sources of Fe and S (Spietz, Payne, Kulkarni, et al. 2022; Spietz, Payne, Szilagyi, and Boyd 2022; Payne, Spietz, and Boyd 2021). These observations were recently extended to show that model sulphate reducers, fermenters and fumarate respirers could also use FeS and FeS_2_ as sources of Fe and S (Boyd and Payne 2025). Further, *Methanosarcina barkeri* was shown to acquire Ni from nickelian pyrite [(Ni,Fe)S_2_] (Spietz et al. 2023). Acquisition of Ni, Fe and S by *M. barkeri* involves reductive dissolution of (Ni,Fe)S_2_ through a yet to be determined mechanism of extracellular electron transfer (EET). Here, we aimed to further expand these studies to examine the factors that shape the Co requirement and mode of Co acquisition in the model methanogens, *Methanosarcina barkeri* strain Fusaro and *Methanococcus maripaludis* strain S2. These organisms represent both more recently diverged Class II/III ( *M. barkeri* ) and the more ancestral Class I ( *M. maripaludis* ) methanogens that occupy different habitats due to their evolutionary histories (Anderson et al. 2009; Bapteste et al. 2005; Lyu and Lu 2018). In this study, we initially examined the influence of Co availability on growth kinetics with different methanogenesis substrates ( *M. barkeri* ) and with different sulphur sources ( *M. maripaludis* ). We then investigated Co acquisition from cobaltoan pyrite [(Co,Fe)S_2_] and whether this was driven by EET, as was previously observed for (Ni,Fe)S_2_.

## 2 Experimental Procedures

### 2.1 Mineral Synthesis

All chemicals used in the mineral syntheses were American Chemical Society (ACS)‐grade or better. All glassware used in synthesis of minerals or in growth experiments was soaked overnight in 10% trace‐metal grade nitric acid (HNO_3_) and was rinsed twice with 18.2 Ω MilliQ water (MQ H_2_O) prior to use. Synthetic pyrite (FeS_2_) was prepared as previously described (Berner 1969) and FeS_2_ that contained cobalt (Co,Fe)S_2_, with a final target Co concentration of 1.67 wt%, was prepared as previously described for (Ni,Co)S_2_ (Spietz et al. 2023). Briefly, for the synthesis of FeS_2_, two anoxic solutions were prepared inside of an anaerobic chamber (Coy Labs, Grass Lake, MI). The first contained 16.7 g FeSO_4_·7H_2_O dissolved in 50 mL MQ H_2_O and the second contained 14.4 g Na_2_S·9H_2_O dissolved in 50 mL MQ H_2_O. For the synthesis of (Co,Fe)S_2_, two solutions were made in anoxic 50 mL MQ H_2_O, one of which contained 15.84 g FeSO_4_·7H_2_O and 0.714 g CoCl_2_·6H_2_O and the second of which contained 14.44 g Na_2_S·9H_2_O. For each synthesis, the two solutions were mixed in a 500 mL bottle for 15 min in the anaerobic chamber, followed by addition of 2.1 g of elemental sulphur (S^0^). The bottle was then sealed with a butyl rubber stopper, removed from the chamber and sparged for 45 min using N_2_ passed over heated (200°C), H_2_‐reduced copper shavings (herein referred to treated N_2_). After sparging, the mixture was incubated for 4 days at 65°C and then for an additional 4 days at 85°C. As a final step to remove unreacted HS^−^, Fe(II), FeS and S^0^, the synthesized nanoparticulate FeS_2_ and (Co,Fe)S_2_ were washed with 1 N HCl four times, washed with boiling 6 N HCl once, washed with MQ H_2_O twice and then washed with > 99.5% acetone three times. Following washing, the synthesized mineral was transferred to an anaerobic chamber and was then washed an additional three times with 0.22 μm‐filtered MQ H_2_O. Following washing, the slurry was centrifuged in sealed tubes at 1000 × *g* for 10 min at 4°C. The pellet was then resuspended in 100 mL sterile, anoxic MQ H_2_O and transferred to a sterile serum bottle that was then capped with a butyl rubber stopper. Triplicate 1 mL subsamples of each mineral slurry were dried under N_2_ gas and weighed to determine the weight to volume ratio (wt/vol) of the slurry.

### 2.2 Synthetic Mineral Characterization

Separate aliquots of synthetic FeS_2_ and (Co,Fe)S_2_ were dried under a stream of 0.22 μm‐filtered N_2_ gas. Roughly 0.1 g of each dried mineral was then subjected to X‐ray powder diffraction (XRD) using a SCINTAG X‐1 system (Eigenmann GmbH, Mannheim, Germany). Aliquots of mineral were also imaged with field emission scanning electron microscopy (FE‐SEM) using a Supra 55VP electron microscope (Zeiss, Thornwood, NY) with a primary electron beam energy of 1 keV at the Imaging and Chemical Analysis Laboratory at Montana State University. Energy dispersive X‐ray spectrometer (EDS) was used to assess metal composition. A third aliquot (~0.1 g) of each dried mineral was dissolved in 40% trace metal free‐HNO_3_ for 72 h at room temperature (~21°C). Dissolved minerals were diluted with MQ H_2_O to 3% HNO_3_ and were then filtered through a 0.22 μm syringe filter. The filtered dissolved mineral solutions were then subjected to atomic absorption (AA) spectroscopy using an Agilent 240 FS instrument (Agilent Technologies, Santa Clara, CA) equipped with Fe and Co lamps using an acetylene/air mixture (11:60 lb in^−2^ partial pressures) as a fuel source. Fe and Co were quantified using standard curves made from 1000 ppm Fe or Co standards (Ricca Chemical Company, Arlington, TX). A fresh 3% HNO_3_ solution in MQ H_2_O was used to prepare each standard dilution series. After dissolving in the 3% HNO_3_ solution, the supernatants were filtered (0.22 μm).

### 2.3 Strains, Growth Media and Cultivation Conditions

#### 2.3.1 *Methanosarcina barkeri*

*M. barkeri* strain Fusaro was obtained from the American Type Culture Collection (ATCC‐BAA‐2329). *M. barkeri* was maintained in low‐salinity base salts medium that contained the following (g L^−1^): NaCl, 1.0; MgCl_2_·6H_2_O, 0.40; NaHCO_3_, 2.0; NH_4_Cl, 0.50; KH_2_PO_4_, 0.15; KCl, 0.50; and CaCl_2_·2H_2_O, 0.10. After boiling base salt medium for 20 min, it was sparged for 1 h with treated N_2_ gas. After sparging, the base salt medium was transferred to an anaerobic chamber and allowed to cool to room temperature (~21°C). After cooling, NaHCO_3_ (2.00 g L^−1^) was added to the medium, followed by adjustment of the pH to 7.0 with anoxic HCl or NaOH. Seventy‐five millilitres of base salt media were added to 165 mL serum bottles and these were sealed with blue butyl rubber stoppers and aluminium crimp caps. Prior to use, butyl rubber stoppers were boiled in MQ H_2_O for 20 min to remove potential contaminants. The headspace of serum bottles was then purged with N_2_:CO_2_ (80:20 vol./vol.) for 20 min. After that, the bottles and their contents were subjected to autoclave sterilization.

After autoclaving, serum bottles and their contents were amended with filter‐sterilized (0.22 μm) anoxic Wolfe's vitamins and trace elements to final concentrations of 10 mL L^−1^ (1% vol./vol.). Wolfe's vitamins contained (g L^−1^): Pyridodoxine HCl, 0.01; thiamine HCl, 0.005; riboflavin, 0.005 g; nicotinic acid, 0.005; calcium D(+) pantothenate, 0.005; biotin, 0.002; and folic acid, 0.002. The trace elements solution contained (g L^−1^): ZnCl_2_, 0.070; MnCl_2_·4H_2_O, 0.100; H_3_BO_3_, 0.006; CoCl_2_·6H_2_O, 0.190; CuCl_2_·2H_2_O, 0.002; NiCl_2_·6H_2_O, 0.024; Na_2_MoO_4_·2H_2_O, 0.036. A separate trace elements solution was made that lacked CoCl_2_·6H_2_O. Autoclaved and anoxic solutions of methanol and acetate were added to final concentrations of 0.5% vol./vol. and 4 mM, respectively. Medium for ferrous iron (Fe(II))‐ and sulphide (HS^−^)‐grown cultures was amended with FeCl_2_·6H_2_O and Na_2_S·9H_2_O to final concentrations of 20 μM and 2 mM, respectively. Medium for Fe(II)/cysteine (Cys)/HS^−^‐grown cultures was amended with FeCl_2_·6H_2_O to a final concentration of 20 μM, L‐cysteine to a final concentration of 2 mM and HS^−^ to a final concentration of 50 μM. Fe and S solutions were anoxic and filter‐sterilized (0.22 μm) and were added 30 min prior to inoculation. All bottles were pressurized with specified gases to 2 atm.

Cultures were incubated at 38°C in the dark and maintained by weekly transfers (10% vol./vol.) of log‐phase cultures into fresh medium containing Fe(II)/Cys/HS^−^ as Fe and S source and acetate and methanol as methanogenesis substrate. Prior to use as inoculum for experiments, cells were washed by first pelleting log‐phase cultures via centrifugation (4696 × *g*, 20 min, 20°C) using sealed 50 mL centrifuge tubes (Globe Scientific, Mahwah, NJ). The cell pellet was resuspended in a sterile and anoxic Fe/Co/S free basal medium for use as inoculum.

#### 2.3.2 *Methanococcus maripaludis*

*M. maripaludis* strain S2 was kindly provided by Dr. Kyle Costa (University of Minnesota). *M. maripaludis* was maintained in base salts medium that contained (g L^−1^): NaCl, 21.98; MgCl_2_·6H_2_O, 5.1; NaHCO_3_, 5.0; NH_4_Cl, 0.50; K_2_HPO_4_, 0.14; KCl, 0.33; and CaCl_2_·2H_2_O, 0.10. The medium was prepared as above and 75‐mL aliquots were dispensed into 165‐mL serum vials. Base salts medium was amended with trace element, vitamin, formate and acetate solutions, each to a final concentration of 1% vol./vol. The trace element solution contained (g L^−1^): ZnCl_2_, 0.0474; MnCl_2_·4H_2_O, 0.0849; nitriloacetic acid, 1.5; CoCl_2_·6H_2_O, 0.100; CuCl_2_·2H_2_O, 0.0683; NiCl_2_·6H_2_O, 0.025; Na_2_MoO_4_·2H_2_O, 0.100; NaSeO_3_, 0.200; Na_2_WO_4_·2H_2_O, 0.100. A separate trace element solution was made that lacked CoCl_2_·6H_2_O and this was used where indicated. The vitamin solution contained (g L^−1^): Pyridoxine HCl, 0.0100; thiamine HCl, 0.0050; riboflavin, 0.0050; nicotinic acid, 0.0050; Ca D(+) pantothenate, 0.0050; biotin, 0.0020; folic acid, 0.0020; and vitamin B_12_, 0.0001. A separate trace vitamin solution without B_12_ was used for where indicated. The vitamins solution was filter‐sterilized (0.22 μm) whereas the trace element, formate (40% wt./vol.) and acetate (1 M) solutions were sterilized individually by autoclaving. All solutions were sparged with treated N_2_ gas. Base salts medium was amended with solutions of FeCl_2_·6H_2_O and HS^−^ to final concentrations of 20 μM and 2 mM, respectively.

Cultures were maintained by twice weekly transfers (10% vol./vol.) of log‐phase cells into fresh medium containing Fe(II)/HS^−^ and formate as methanogenesis substrate. All cultures were incubated in the dark at 37°C and were pressurized to 2 atm with 80:20 N_2_:CO_2_. Prior to use as inoculum for experiments, cells were washed as described above. The cell pellet was resuspended in a sterile and anoxic Fe/Co/S‐free basal medium for use as inoculum.

### 2.4 Influence of Cobalt and Methanogenesis Substrate on Growth of *M. barkeri*

Cultures of *M. barkeri* were grown as described above in the absence of a Co source to initiate Co limitation. Co‐limited *M. barkeri* cultures were provided with Fe(II)/cysteine/HS^−^ as Fe and S sources and the methanogenesis substrate was either methanol and acetate, methanol only or H_2_/CO_2_. Methanol and acetate were provided at final concentrations of 0.5% vol./vol. and 4 mM, respectively. Methanol was added to a final concentration of 0.5% vol./vol. in cultures grown with methanol only. For H_2_/CO_2_‐grown cultures, the N_2_ headspace was replaced by purging with an 80/20% mixture of H_2_/CO_2_ to a final pressure of 2 atm; these cultures were not amended with acetate or methanol. The base salts medium was amended with trace elements containing Co (Co‐replete condition) or with trace elements lacking Co (Co‐deplete condition).

### 2.5 Influence of Cobalt and Sulphur Source on Growth of *M. maripaludis*

Cultures of *M. maripaludis* were grown as described above but in the absence of a Co source to initiate Co limitation. Co‐limited *M. maripaludis* cultures were provided with formate as methanogenesis substrate and 20 μM Fe(II) as Fe source. The S source was either HS^−^, cysteine or thiosulphate and cysteine. For cysteine‐grown cultures, L‐cysteine was added to a final concentration of 1 mM. For thiosulphate and cysteine‐grown cultures, thiosulphate and L‐cysteine were added to final concentrations of 1 mM each. The base medium was amended with trace elements containing Co (Co‐replete) or with trace elements lacking Co (Co‐deplete).

### 2.6 Monitoring Methane Production and Growth

Subsamples of the headspace of cultures were collected with a syringe and stopcock flushed with N_2_. The subsamples of headspace gas were then diluted in ultra‐high purity N_2_ using pre‐evacuated and N_2_‐purged CaliBond bags (Calibrated Instruments Inc., Manhasset, NY). Eight millilitres of headspace gas was subjected to gas chromatography using a SRI 8610C gas chromatograph (SRI Instrument Company Inc., Torrance, CA) equipped with a Hayesep DB 100/120 packed column (Valco Instrument Company Inc., Houston, TX). The temperature of the injector valve was set to 55°C and the oven was set to 44°C. Helium was used as the carrier gas and CH_4_ was measured using a flame‐ionization detector that was set at a temperature of 156°C. CH_4_ peak area was converted to concentration using a dilution series of a certified CH_4_ standard (EGAS Depot, Nampa, ID). The production of HS^−^ in cultures provided with FeS_2_ or (Co,Fe)S_2_ was measured using the methylene blue assay (Fogo and Popowsky 1949). Methylene blue was quantified by measuring absorbance at 670 nm with a Genesys 10S Vis Spectrophotometer (Thermo Scientific, Waltham, MA) and using freshly prepared Na_2_S standards. Henry's law was used to calculate total sulphide (aqueous HS^−^ and gaseous H_2_S) as previously described (Boyd et al. 2007).

Growth of *M. barkeri* and *M. maripaludis* was monitored by quantification of genomic DNA as a proxy for cell production to avoid underestimates of growth in the presence of minerals or due to cell aggregation. To quantify DNA, 1 mL subsamples of suspended *M. barkeri* or *M. maripaludis* cultures (including minerals, when present) were collected during growth via sterile syringe and needle, transferred to sterile microfuge tubes, centrifuged at 20,000 × *g* for 20 min at 4°C and then the supernatant was decanted. After centrifugation, the cell pellet was resuspended in a lysis buffer solution containing 489 μL sodium phosphate buffer (MP Biomedicals, Irvine, CA, United States) and 61 μL MT buffer (MP Biomedicals). The mixture was then gently stirred before freezing three times at −80°C followed by heating to 60°C. The mixture was then transferred to a 2 mL screw cap bead beating tube (MP Biomedicals) and homogenized using a bead beater (Biospec Products, Bartlesville, OK, United States) for 40 s at room temperature (~23°C). To separate DNA from cell and mineral debris, the tube was centrifuged for 15 min at 14,000 × *g* at 4°C. The concentration of DNA was quantified using a Qubit HS Double‐Stranded DNA kit and Qubit fluorimeter (Invitrogen, Carlsbad, CA). Genome copy numbers were calculated from DNA concentrations and the predicted mass of the genome.

### 2.7 Quantifying the Metal Content of Biomass

Biomass from *M. barkeri* and *M. maripaludis* cultures was analysed for Co, Fe and Ni content by harvesting cultures at both early and mid‐log phases via centrifugation (4696 × *g* for 20 min at 4°C). Early‐ and mid‐log phases were defined as the initiation of exponential growth and the time of fastest growth, identified from growth curves (Figures 1 and 2). Metal contents were only measured on cultures provided with soluble sources of Co, Fe and S. The supernatant was removed and cells were resuspended in 1 mL of sterile, anoxic base salts medium that lacked trace elements/vitamins, sources of Fe and S, and methanogenesis substrates but that contained 1 mM nitrilotriacetic acid (NTA) to facilitate removal of unassimilated metals. The cell pellet was gently vortexed several times over a period of 1 min and then cells were pelleted again via centrifugation (4696 × *g* for 20 min at 4°C), resuspended in a minimal amount of sterile MQ H_2_O and transferred to a pre‐weighed, sterile 2 mL screw‐top tube. Cells were dried on a heat block at 70°C overnight and then weighed again to determine dry weight. The dried cell pellets were dissolved using 10% trace metal‐free HNO_3_ in a 98°C heat block with periodic vortexing. Dissolved biomass was diluted with 0.75 mL of 0.22 μm‐filtered MQ H_2_O and metal contents were determined via AA spectroscopy (as described above) or inductively coupled plasma emission‐mass spectrometry (ICP‐MS, as described below).

**FIGURE 1:**
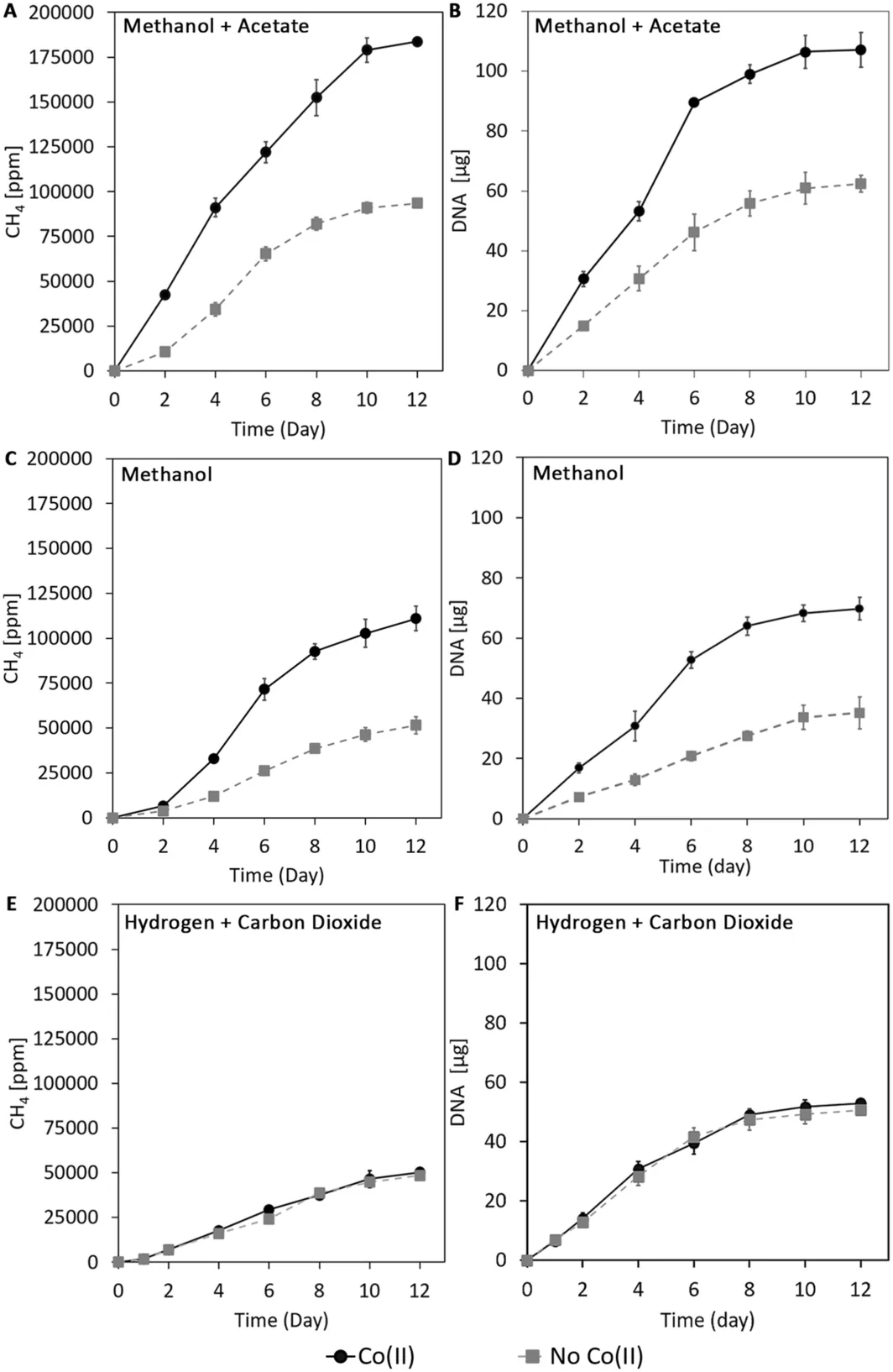
Influence of methanogenesis substrate and the availability of cobaltous cobalt (Co(II)) on the production of methane (CH_4_; A, C, E) and DNA as a proxy for biomass (B, D, F) in cultures of *Methanosarcina barkeri* Fusaro. Cultures were provided with methanol plus acetate (A, B), methanol (C, D) or hydrogen plus carbon dioxide (E, F) as methanogenesis substrates. Cultures were provided with ferrous iron Fe(II), cysteine (Cys) and sulphide (HS^−^) as iron and sulphur sources. Fe(II), Cys and HS^−^ were added to final concentrations of 20 μM, 2 mM and 50 μM, respectively; cobaltous cobalt (Co(II)) was added to a final concentration of 2 μM where indicated. The data shown are the mean and standard deviation of triplicate cultures.

**FIGURE 2:**
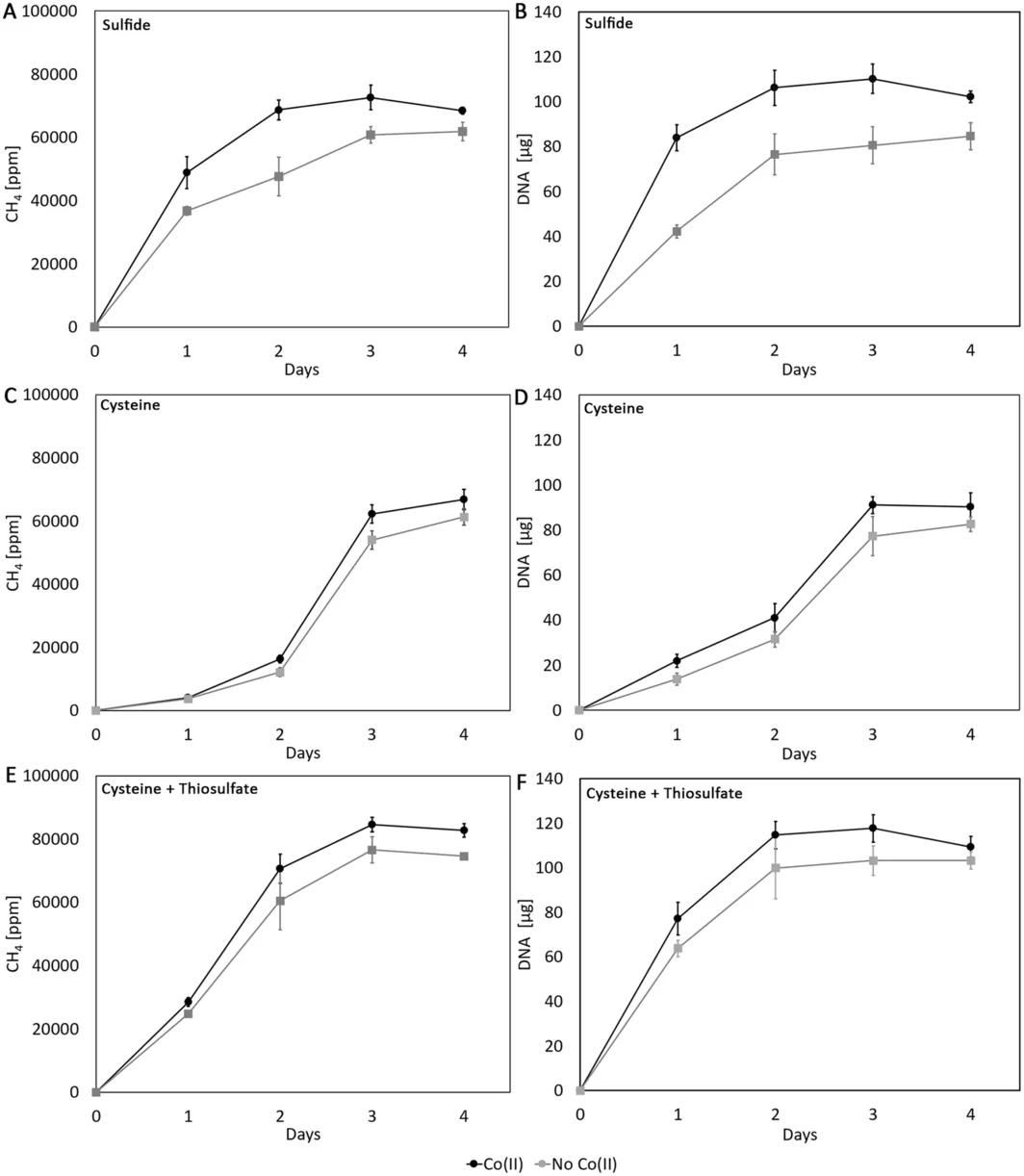
Influence of sulphur source and availability of cobaltous cobalt (Co(II)) on the production of methane (CH_4_; A, C, E) and DNA as a proxy for biomass (B, D, F) in cultures of *Methanococcus maripaludis* S2 provided with formate as methanogenesis substrate. Ferrous iron was added to a final concentration of 20 μM and Co(II) was added to a final concentration of 2 μM, where indicated. Cultures were provided with either sulphide (2 mM final concentration), cysteine (1 mM) or cysteine and thiosulphate (1 mM each). Data shown are the mean and standard deviation of triplicate cultures.

### 2.8 Determining Growth on FeS_2_ and (Co,Fe)S_2_ Minerals

Co‐limited *M. barkeri* and *M. maripaludis* cells were examined for their ability to utilize mineral sources of Fe, S and Co. Cultures of *M. barkeri* were grown in base salts medium with methanol and acetate, as described above, with soluble Fe(II), cysteine and HS^−^ at final concentrations of 20 μM, 2 mM and 50 μM, respectively, in medium with soluble Co(II) (Co‐replete condition) or without soluble Co(II) (Co‐deplete condition), where indicated. Synthetic FeS_2_ or (Co,Fe)S_2_ nanoparticles were added to a final concentration of 2 mM Fe and cultures were incubated as described above. Cultures of *M. maripaludis* were grown with formate, as described above, with soluble Fe(II) and HS^−^ at final concentrations of 20 μM and 2 mM, respectively, in base salts medium with soluble Co(II) (Co‐replete condition) or without soluble Co(II) (Co‐deplete condition), where indicated.

Samples of *M. maripaludis* cultures grown with or without (Co,Fe)S_2_ were collected during exponential phase for visualization by FE‐SEM. Aliquots of cultures were collected in an anaerobic chamber and were fixed overnight at 4°C with 2.5% glutaraldehyde. Fixed cells were washed with sterile base salts medium and collected on gold‐sputtered 0.2‐μm filters with a microfiltration and vacuum apparatus. The cells were dehydrated for 5 min at each step in a series of ethanol baths (50%, 70%, 80%, 90%, 95% and 100% ethanol) and air dried at 4°C prior to imaging. Filters were affixed to FE‐SEM stages with double‐sided carbon tape and imaged with FE‐SEM, as described above.

### 2.9 Assessing Mechanisms of Mineral Reduction

Dialysis membranes were used to assess the requirement for direct access to (Co,Fe)S_2_ mineral for reduction, solubilization and use as a Fe/Co/S source in cultures of *M. barkeri* and *M. maripaludis* . A 10 cm section of Spectra/Por dialysis tubing with 12–14 kDa pore sizes (Spectrum Labs Inc., Rancho Dominguez, CA) was used to physically isolate (Co,Fe)S_2_ from cells during growth. Prior to their use, dialysis membrane sections were washed to remove preservatives and potential contaminants. Ten centimetre lengths of tubing were soaked for 30 min in MQ H_2_O at 65°C before decanting and rinsing with 0.22 μm‐filtered MQ H_2_O in the first step of washing dialysis membranes. The pre‐cut membranes were then transferred to a 50% ethanol solution and these were incubated at 65°C overnight. Washed dialysis tubes were then transferred to 0.22 μm‐filtered MQ H_2_O and all further manipulations were performed in a UV‐treated laminar flow hood using gloves and forceps treated with ethanol. One end of the dialysis tubing was sealed using pre‐washed and treated monofilament line (Berkeley, Columbia, SC) and then (Co,Fe)S_2_ was added to achieve a final concentration of 2 mM Fe once added to culture bottles. The tubing containing (Co,Fe)S_2_ was brought into an anaerobic chamber and the other end of the tube was tied with monofilament line. In the final step, the outsides of each tube were washed with sterile, anoxic MQ H_2_O and placed into 165 mL serum bottles that had been pre‐autoclave sterilized. These bottles contained 75 mL of base salts medium prepared as described above. The bottles were then sparged with 0.2 μm‐filtered N_2_:CO_2_ (80:20 vol./vol.) to remove H_2_ from the atmosphere of the anaerobic chamber and were then pressurized to 2 atm.

### 2.10 Quantifying Abiotic Dissolution of (Co,Fe)S_2_

The dissolution of synthetic (Co,Fe)S_2_ was evaluated in 35 mL of anoxic, autoclaved MQ H_2_O in 165 mL autoclave serum bottles. (Co,Fe)S_2_ was added to a final concentration of 2 mM Fe and was incubated for 48 h at 38°C in the dark under a 100% vol./vol. N_2_ or H_2_ headspace (2 atm). Subsamples of reactor contents were filtered (0.22 μm) after incubation, acidified and subjected to ICP‐MS. Samples were acidified with HNO_3_ and analysed on a model 7800 ICP‐MS equipped with an SPS4 autosampler (Agilent Technologies). Metal concentrations were determined using standard curves generated from serial dilution of a commercially available environmental calibration standard (CPI International, Santa Rosa, CA). The detection limit was determined as either 3× the standard deviation of the calibration blank or the blank equivalent concentration based on whichever was greater. An internal standard mix (Agilent Technologies) was added to the samples using a T junction immediately upstream of the nebulizer. The ICP‐MS parameters were auto‐tuned using an ICP‐MS tuning solution (Agilent Technologies). In some cases, samples were diluted with a 2% HNO_3_, 0.5% HCl solution to either bring analyte response into the linear range of the instrument or to reduce salt‐based signal suppression.

### 2.11 Cobamide Quantification

Cobamides were extracted from *M. barkeri* biomass using a previously described method (Carkeet et al. 2006). Cultures were grown with methanol and acetate as methanogenesis substrates and with Fe(II)/Cys/HS^−^ as Fe and S sources, as described above, in the presence (Co‐replete condition) or absence (Co‐deplete condition) of Co(II) at a final concentration of 2 μM. Cultures were also grown with (Co,Fe)S_2_ as the sole source of Co, Fe and S. Briefly, mid‐log phase cells were pelleted by centrifugation (20 min, 4696 × *g*, 4°C) and the supernatant was decanted. Cell pellets were resuspended in 30 mL of anoxic, sterile base salts medium that lacked trace elements/vitamins, sources of Fe and S and methanogenesis substrates and were again pelleted by centrifugation (60 min, 4696 × *g*, 4°C). The washed pellets were resuspended in 5 mL of methanol containing sodium cyanide (NaCN) at a final concentration of 50 mg mL^−1^. The sample was then placed in a 60°C water bath for 6 h with intermittent vortex mixing. Following an additional 6 h incubation at 60°C, the samples were centrifuged (1 h, 20,000 × *g*, 4°C) and the supernatants were collected and evaporated to dryness in a chemical fume hood under a stream of 0.2 μm‐filtered N_2_ gas. Dried samples were resuspended in MQ water and passed through 0.2 μm filters to remove insoluble material. Cobamides were detected by measuring absorbance at 361 nm using a Genesys 10S Vis Spectrophotometer (ThermoFisher). Peak amplitude was converted to concentration using cyanocobalamin (Acros Organics, New Jersey, USA) as a standard.

## 3 Results and Discussion

### 3.1 Proxies for Monitoring Growth

Methanogens in natural and laboratory systems are classically monitored for respiratory activity by the production of CH_4_. However, methods to track growth are often complicated by low biomass yields (Boyd et al. 2010), morphological pleiomorphy (Jones et al. 1983), differences in cell behaviour (e.g., clumping (Sowers et al. 1993)) or even genome ploidy (Hildenbrand et al. 2011; Malandrin et al. 1999). We compared cell counts, CH_4_ production and DNA concentration to identify an accurate, yet high‐throughput method to monitor the development of *M. barkeri* and *M. maripaludis* cultures. Cultures of *M. barkeri* and *M. maripaludis* were grown as described above with methanol and acetate or with formate as the methanogenesis substrates, respectively.

#### 3.1.1 M. barkeri

Despite notable clumping of cells, direct cell counts resulted in an estimated generation time of 11.3 h (Figure S1A). Culturing conditions used in this study resulted in similar rates of cell and CH_4_ production, as both hit asymptotes at roughly the same time during incubation (Figure S1B). The concentration of DNA from these cultures doubled in 20.8 h and produced curves that more closely resembled CH_4_ yields (Figure S1C). Assuming that DNA recovery was 100% efficient, the number of genome copies per cell never exceeded one, which contrasts with three copies per cell during slow growth in the related strain *Methanosarcina acetivorans* (Hildenbrand et al. 2011). Reconciling these differences, it is possible that cell concentrations determined here underrepresented true cell concentrations due to clumping of these cells under low‐salt conditions (Spietz et al. 2023) or that DNA recovery from cells was not 100% efficient. Thus, we conclude that CH_4_ and DNA are more reliable methods for quantifying growth in the current cultivation conditions and these offer the additional benefit of being both rapid and high throughput.

#### 3.1.2 M. maripaludis

Direct cell counts produced a typical sigmoidal growth curve of *M. maripaludis* (Figure S1D). Cultures rapidly entered exponential growth and reached a minimum generation time of 5.8 h. After growing to a maximum density of 2.8 × 10^8^ cells mL^−1^, cultures entered stationary phase but continued to produce CH_4_ (Figure S1E). Indeed, CH_4_ concentrations increased by an additional 13%, presumably to meet continued maintenance energy needs. Monitoring cultures of *M. maripaludis* using CH_4_ production can therefore be inaccurate at late growth stages, depending on the culturing conditions. On the other hand, DNA concentrations mirrored the concentration of cells and estimated a similar generation time of 6.1 h (Figure S1F). While *M. maripaludis* is notably polyploid (Hildenbrand et al. 2011), between 1 and 2 genome copies per cell were estimated here throughout all growth stages. Nonetheless, the similarity between DNA concentration and cell production suggests DNA as a suitable, high‐throughput method for monitoring *M. maripaludis* cultures.

### 3.2 Influence of Co Availability and Methanogenesis Substrate on Activity and Growth

#### 3.2.1 M. barkeri

The dependency of *M. barkeri* on Co was evaluated under methylotrophic (methanol and acetate or methanol only) and hydrogenotrophic (H_2_/CO_2_) growth conditions, with Fe(II) as Fe source and Cys/HS^−^ as S sources. Production of CH_4_ and DNA were highest in cultures provided with Co and with methanol and acetate as methanogenesis substrates (Figure 1A,B), followed by those provided with methanol only (Figure 1C,D) and those provided with H_2_/CO_2_ (Figure 1E,F). The enhanced growth in methanol and acetate‐ versus methanol only‐grown cultures is likely attributable to the preferential assimilation of acetate into biomass, allowing for increased flux of methanol through methanogenesis energy conservation pathways involving Mtr and not acetoclastic methanogenesis. This is based on (1) calculations indicating that > 80% of supplied methanol was still available for use in methanol and acetate‐grown cultures and (2) the higher thermodynamic yield of methanogenesis via methanol fermentation (ΔG^0^′ = −106.5 kJ mol^−1^) than acetate fermentation (ΔG^0^′ = −36.0 kJ mol^−1^) (Thauer 1998). Even if acetate fermentation was occurring and CODH functioned as the point of acetate entry into methanogenic pathway, the bidirectionality of CODH (Ragsdale and Kumar 1996) that is already being produced for biomass synthesis would not necessarily increase the Co demand in cells. CH_4_ and DNA production in Co‐deplete cultures grown with methanol and acetate or methanol only were roughly half of those provided with Co. However, the kinetics of CH_4_ and DNA production by cultures grown with H_2_/CO_2_ in Co‐replete and Co‐deplete medium were nearly identical. Hydrogenotrophic, methylotrophic and acetoclastic methanogenesis all require the cobamide‐dependent, ion pumping Mtr (Gärtner et al. 1993) and cobamide‐dependent CODH (Ragsdale and Kumar 1996; Svetlitchnaia et al. 2006), with methylotrophic methanogenesis requiring an additional cobamide‐dependent methyltransferase, Mta, to initiate methanogenesis by the formation of methyl‐Coenzyme M (CH_3_‐CoM) from methanol (Harms and Thauer 1996). Co‐deficient Mta would be expected to severely limit energy conservation via decreased flux of CH_3_‐CoM for methyl CoM reductase driven formation of heterodisulphide CoM‐S‐S‐CoB (reductive direction) and Co‐dependent ion pumping methyltransferase (oxidative direction), as well as decreased flux for biomass production. The difference in growth response to Co depletion between methylotrophic and hydrogenotrophic conditions suggests that cells require more Mta than Mtr or that cells prioritize cobamide loading to Mta relative to Mtr.

The dependency of *M. barkeri* cultures on Co was also evident in the amount of the metal in biomass (Table 1). Co‐replete cultures contained the same amount of Co regardless of methanogenesis substrate, consistent with previous reports of the metal content of this strain under various culturing conditions (Scherer et al. 1983). Further, the amount of cellular Co increased from early‐ to mid‐log phase. Similarly, cells grown with any of the methanogenesis substrates evaluated were significantly depleted of Co when it was restricted from the medium, in particular in H_2_/CO_2_‐grown cultures. This suggests that the low amount of Co associated with H_2_/CO_2_ biomass in Co‐deplete culture conditions, which grew as well as Co‐replete conditions, is sufficient to meet Co biosynthesis demands. If it is assumed that all Co in the medium was incorporated into biomass, the starting soluble concentration would be ~55 nM. While ICP‐MS measurements of the base media and non‐metal components did not detect contaminant Co within the standard curve (< 6 nM), it is possible that Co was carried over with the inoculum as some methanogens are highly resistant to Co starvation (Stupperich and Kräutler 1988). Low Co concentrations like this were found to limit growth of *Methanothermobacter thermoautotrophicus* when grown with H_2_/CO_2_ (Taylor and John Pirt 1977) and such concentrations are well below those typically used to culture other methanogens (Glass and Orphan 2012). These data suggest that *M. barkeri* , when grown under Co‐replete conditions, accumulates approximately 20‐ to 40‐fold more Co than is necessary to support hydrogenotrophic methanogenesis, possibly to support metabolic flexibility should more favourable methanogenesis substrates become available. Interestingly, limitation of Co decreased growth in methanol only‐grown cells to levels similar to the H_2_/CO_2_‐grown cells, but levels of growth with methanol and acetate were higher. If acetate is primarily supporting C‐assimilation, then the enhanced growth of cells provided with methanol and acetate may indicate that Co limitation is primarily impacting methanogenesis and not biosynthesis pathways. These varied impacts by Co depletion are surprising given that the amount of Co recovered in biomass in all growth conditions was a small fraction (< 0.14%) of the total Co available in the growth medium when Co was provided.

**TABLE 1:** The effect of methanogenesis substrate, sulphur source and cobalt (Co) availability on the amount of Co, iron (Fe) and nickel (Ni) in biomass of *Methanosarcina barkeri* Fusaro or *Methanococcus maripaludis* S2 in early or middle log phase cultures.

*M. barkeri*	Methanogenesis substrate	Growth phase	Cobalt (±)	Co		Fe		Ni	
				Avg.	Std. dev.	Avg.	Std. dev.	Avg.	Std. dev.
	Methanol/Acetate	Early log	+Co	28	10	464	42	26	6
			−Co	8	1	221	37	23	6
		Mid log	+Co	34	6	496	60	27	9
			−Co	7	2	229	36	31	25
	Methanol	Early log	+Co	22	1	503	3	108	3
			−Co	7	4	236	67	67	20
		Mid log	+Co	27	7	446	41	106	42
			−Co	5	2	302	43	159	76
	H_2_/CO_2_	Early log	+Co	36	4	251	12	24	3
			−Co	2	0	251	40	38	4
		Mid log	+Co	41	7	270	14	61	2
			−Co	1	1	381	36	71	13

*M. maripaludis*	Sulphur source	Growth phase	Cobalt (±)	Co		Fe		Ni	
				Avg.	Std. dev.	Avg.	Std. dev.	Avg.	Std. dev.
	Sulphide	Early log	+Co	557	98	1846	310	137	18
			−Co	16	13	2657	78	152	10
		Mid log	+Co	926	351	3232	560	192	36
			−Co	31	11	3335	123	223	26
	Cysteine + Thiosulphate	Early log	+Co	151	55	1544	331	24	10
			−Co	24	34	1138	184	9	5
		Mid log	+Co	175	23	1295	458	18	7
			−Co	27	26	2389	246	12	5
	Cysteine	Early log	+Co	114	29	1584	186	13	5
			−Co	2	3	928	134	4	4
		Mid log	+Co	117	36	1457	85	13	5
			−Co	7	5	1031	58	8	1

*Note:* *M. barkeri* was grown with cysteine and sulphide as S source with specified methanogenesis substrates. *M. maripaludis* was grown with formate as methanogenesis substrate with specified sulphur sources. The average and standard deviation of metal concentrations (ppm) are reported for triplicate cultures normalized to dry weight biomass.

The amount of Fe and Ni in *M. barkeri* biomass also varied as a function of methanogenesis substrate, stage of growth and whether Co was provided to cultures (Table 1). Methanol and acetate‐ or methanol only‐grown cells provided with Co had significantly more Fe than H_2_/CO_2_‐grown cells, but only in early‐log phase cultures. Mid‐log phase H_2_/CO_2_‐grown cells with Co added had more Fe than methanol and acetate‐ or methanol only‐grown cells. Interestingly, cells grown with methanol and acetate had less Fe when Co was omitted from the medium than when Co was added, regardless of growth stage. However, H_2_/CO_2_‐grown cells had similar (early‐log) or more (mid‐log) Fe in biomass when Co was omitted than when Co was added. The amount of Ni in methanol and acetate‐grown cells was similar regardless of whether Co was added and this was independent of growth stage. Methanol only‐grown cells had roughly twice the amount of Fe than methanol and acetate‐grown cells and this did not vary with growth stage or if medium was supplemented with Co. However, H_2_/CO_2_‐grown cells had slightly higher amounts of Ni when Co was omitted from the growth medium. These observations suggest potential interplay among pathways of Co, Fe and Ni acquisition and use in *M. barkeri* . The amount of Fe and Ni recovered in biomass never exceeded 0.6 or 4.5%, respectively, of the total amount of those metals when provided in the growth medium.

#### 3.2.2 M. maripaludis

The dependency of *M. maripaludis* on Co was evaluated by quantifying production of CH_4_ and DNA in formate‐grown cultures provided with Fe(II) as Fe source and HS^−^, cysteine or cysteine and thiosulphate as S sources. While *M. maripaludis* can also grow with H_2_/CO_2_ as methanogenesis substrate (Abdul Halim et al. 2024), only formate‐grown cultures were evaluated due to abiotic reactions between H_2_ and FeS_2_ (Spietz, Payne, Kulkarni, et al. 2022), which would confound downstream experiments with this mineral or (Co,Fe)S_2_ (described below). Formate‐grown *M. maripaludis* cultures produced more CH_4_ and DNA when provided with Co than when Co was omitted from the medium regardless of the S source provided, although the difference was only statistically significant (*p*‐value < 0.05) in cultures grown with HS^−^ (Figure 2A,B). HS^−^‐grown cultures also had shorter lag phases for CH_4_ and DNA production when compared to those grown with cysteine (Figure 2C,D) or cysteine and thiosulphate (Figure 2E,F). However, the highest amount of CH_4_ and DNA produced in cultures was observed in cultures grown with cysteine and thiosulphate, regardless of whether medium was amended with Co.

Early‐ and mid‐log phase *M. maripaludis* accumulated 5.5 to 7.9 and 3.7 to 4.9 times more Co when grown with HS^−^ than when grown with cysteine or cysteine and thiosulphate, respectively (Table 1). However, the omission of Co from the medium decreased Co in *M. maripaludis* cells to the same amount regardless of the S source provided. And yet Co omission only had a significant phenotypic impact on HS^−^‐grown cells. The resilience of cysteine‐only and cysteine and thiosulphate‐grown cells to Co limitation suggests that the trace amount of Co provided (as impurities) and assimilated is sufficient to meet their biosynthetic demands but is insufficient in cells grown in sulphidic conditions, which points to HS^−^ affecting Co availability, as discussed below. While the cells were washed with NTA (1 mM final concentration) to remove loosely bound metals from the surfaces of cells, the amount of Co associated with biomass in Co‐replete grown cultures may suggest non‐specific adsorption of Co to functional groups that have a higher affinity for the metal than NTA. Nonetheless, the total amount of Co associated with culture biomass never exceeded 6.4% of the total Co added to medium.

The inclusion or exclusion of Co from growth medium also affected the amount of Fe and Ni in biomass of *M. maripaludis* when grown with HS^−^, cysteine or cysteine and thiosulphate but in different ways. Cells grown with cysteine or cysteine and thiosulphate accumulated less Fe and Ni when Co was omitted, while cells grown with HS^−^ accumulated greater amounts of Fe and Ni regardless of the presence of Co. Like *M. barkeri* , this may suggest interplay among the acquisition and usage of Co, Fe and Ni in *M. maripaludis* cells that is responsive to the S source provided. For instance, the hyperaccumulation of Fe and Ni, as well as Co when available, by HS^−^‐grown cells may be due to their thiophilic nature. All three metals rapidly react with HS^−^ to generate metal‐sulphide aqueous clusters (Rickard and Luther 2006) leading to a several fold decrease in soluble metal concentrations (Mazumder et al. 1987). Previous studies have observed intracellular Fe hyperaccumulation as a detoxification mechanism in methanogen cells growing on iron sulphides as the sole source of Fe and S (Payne, Shepard, et al. 2021) and in hyperthermophilic *Pyrococcus* cells reducing S^0^ (Vaccaro et al. 2017). In the case of the former, this observation was hypothesized to be due to formation of soluble, neutrally charged FeS_aq_ during reduction of FeS_2_ that can readily transit the membrane (Payne, Shepard, et al. 2021). Since methanogens require roughly an order of magnitude more S than Fe for biosynthesis (Scherer et al. 1983), it was hypothesized that excess Fe was sequestered as an insoluble secondary mineral (Payne, Shepard, et al. 2021). It is possible that Co and Ni are similarly being accumulated intracellularly due to passive diffusion of CoS_aq_ and NiS_aq_ and sequestration of excess metal as secondary minerals. Additional spectroscopic studies are needed to assess secondary mineral formation in methanogens.

The reactivity of Co with HS^−^ and limited reactivity between Co and thiosulphate or cysteine, may explain the observed differences in growth when Co is absent. While trace amounts of Co in deplete medium may support growth with thiosulphate or cysteine, the presence of 2 mM HS^−^ would be expected to precipitate the available metal (Larson et al. 2024). Extending the logic of Luther and Rickard (see Fig. 28 in (Rickard and Luther 2007)) from Fe to Co, the more than three orders of magnitude excess of HS^−^ would be expected to keep Co precipitated as an insoluble phase rather than for it to equilibrate as potentially bioavailable CoS_aq_ clusters. As such, the bioavailable Co in equilibrium with HS^−^ in deplete medium is low enough to moderately impair the growth of *M. maripaludis* while the 4.2 μM provided in replete medium is high enough to overcome biological limitation. Despite possible differences in metal precipitation in connection to the provided S sources, the amount of Fe and Ni associated with biomass never exceeded 4.1 or 6.2%, respectively, of the total amount of those metals in the growth medium.

### 3.3 Influence of Soluble Versus Mineral Sources of Co on Activity and Growth

#### 3.3.1 Characterization of Synthesized Minerals

FeS_2_ nanoparticles were synthesized via the sulphur oxidation method (Berner 1969), which has previously been shown to be effective for synthesis of FeS_2_ with both Co and Ni impurities (Swanner et al. 2019; Spietz et al. 2023). FeS_2_ with target Co concentration of 0.0% and 1.67% w/w were synthesized, the latter of which by replacing an equivalent amount of Fe (as FeSO_4_) with Co (as CoCl_2_) in the synthesis reaction. Analysis of the FeS_2_ and (Co,Fe)S_2_ product by acid dissolution and AA spectroscopy indicated final Co concentrations of 0.0 wt% (detection limit of 0.2 ppm) and 2.24 wt% and final Fe concentrations of 45.60 wt% and 42.70 wt%, respectively (Table 2). XRD analysis indicated a near perfect match of synthetic FeS_2_ spectra to that of reference FeS_2_ (Figure S2A). The same major XRD peaks attributed to FeS_2_ were identified in synthetic (Co,Fe)S_2_ alongside minor peaks potentially attributable to cobalt oxide (Co_3_O_4_), cobaltkieserite [Co(SO_4_)(H_2_O)], cobaltpentlandite (Co_9_S_8_), goethite (FeO(OH)) and elemental sulphur (S^0^). Semi‐quantitative mineral analyses based on each spectra indicate the FeS_2_ synthesis to be 100 wt% FeS_2_ whereas that of (Co,Fe)S_2_ indicates a predominance of FeS_2_ (77.2 wt%) followed by Co_3_O_4_ (12.0 wt%), Co(SO_4_)(H_2_O) (5.4 wt%), Co_9_S_8_ (2.5 wt%), FeO(OH) (2.0 wt%) and S^0^ (1.0 wt%) (Figure S2B). It should be noted that, aside from FeS_2_, none of the accessory mineral assignments had 100% matches. Generally speaking, the XRD spectrum of synthetic (Co,Fe)S_2_ synthesized herein was similar to previously reported syntheses, including a major reflection at 40^°^ present in (Co,Fe)S_2_ that was not in FeS_2_ (Swanner et al. 2019). FE‐SEM revealed the primary morphology of (Co,Fe)S_2_ to be ~1 μm diameter framboids that aggregated to form larger clusters (Figure S3A). EDS revealed a near uniform distribution of Co, Fe and S in the clusters of framboids (Figure S3B–D).

**TABLE 2:** The weight percent (wt %) of iron and cobalt in laboratory‐synthesized pyrite (FeS_2_) and cobaltian pyrite [(Co,Fe)S_2_] with a target cobalt concentration of 1.67 wt%.

Mineral	Cobalt (Co)	Iron (Fe)
(Co,Fe)S_2_	2.24 ± 0.07	42.70 ± 0.82
FeS_2_	ND	45.60 ± 1.20

Abbreviation: ND, not detected.

Prior to experimentation, the abiotic dissolution of (Co,Fe)S_2_ was examined following 96 h incubation at 38°C in 0.22 μm‐filter sterilized MQ water, in reactors containing a headspace of N_2_ or H_2_. Previous studies have shown that headspace H_2_ at concentrations as low as 10% vol/vol drive the abiotic reduction of FeS_2_ (Spietz, Payne, Kulkarni, et al. 2022). In reactors with a N_2_ headspace, only 2.8 μM (0.14 μmol total) of HS^−^ was released over the incubation period, indicating that the mineral was stable in the absence of a strong reductant (e.g., H_2_). Twenty nanomolar Co (0.6 nmol) was released into solution from (Co,Fe)S_2_, which is similar to the amount of Ni (19 nM, 0.7 nmol) solubilized from (Ni,Fe)S_2_ under similar incubation conditions (Spietz et al. 2023). The amount of Co passively dissolved from (Co,Fe)S_2_ is similar to what was estimated (~55 nM) to support hydrogenotrophic methanogenesis in *M. barkeri* when grown in the absence of Co (Figure 1). In contrast, 651 μM (33 μmol total) of HS^−^ and 41 nM Co (1.2 nmol) was released from (Co,Fe)S_2_ when incubated in the presence of H_2_ (100% H_2_ headspace). Notably, the concentration of free HS^−^ in solution (651 μM) would have limited the solubility of Co, given the high affinity of HS^−^ and Co (Dehaine et al. 2021; Irving and Williams 1948; Moore et al. 2018; Krupka and Serne 2002). Indeed, previous studies of the abiotic reduction of (Ni,Fe)S_2_ by H_2_ failed to detect Ni release into solution, which was hypothesized to be due to HS^−^‐promoted precipitation of the metal to form aggregates/particles that were too large to pass through a 0.22‐μm filter prior to analysis (Spietz et al. 2023). This suggests that the amount of Co released during the reductive dissolution of (Co,Fe)S_2_ is much greater than observed. The inclusion of trace metal impurities during the formation of FeS_2_ disrupts its stability (Swanner et al. 2019) and may make those minerals more reactive to reduction (Spietz, Payne, Kulkarni, et al. 2022). It is suspected that reduction of the persulphide in (Co,Fe)S_2_ is likely to further disrupt its stability leading to greater solubilization of Co. Further studies are needed to assess the reactivity and solubility of FeS_2_ that contains trace metal impurities (e.g., (Co,Fe)S_2_ or (Ni,Fe)S_2_). This is especially important considering the ubiquity of trace metal impurities in natural FeS_2_ deposits (Herazo et al. 2021; Deditius et al. 2011; Rieger et al. 2023).

#### 3.3.2 M. barkeri

Cultures of methanol and acetate‐grown *M. barkeri* were examined for their ability to use (Co,Fe)S_2_ as a source of Fe, S and Co. Cells grown in Co deplete medium with FeS_2_ produced less DNA and CH_4_, consistent with Co limitation (Figure 3A,B). Despite having no more than 6 nM of Co, these cells produced HS^−^ at levels (< 7 μmol total or < 88 μM) similar to those of *M. barkeri* when grown with FeS_2_ (Spietz, Payne, Kulkarni, et al. 2022; Payne et al. 2023), indicating that Co limitation is not impairing the ability to reductively dissolve the mineral (Figure 3C). However, (Co,Fe)S_2_ readily supported the growth of *M. barkeri* . Amendment of (Co,Fe)S_2_‐grown cultures with additional Co had no additive effect on growth. Further, (Co,Fe)S_2_‐grown cultures produced amounts of HS^−^ that are indicative of mineral reduction coupled to dissolution and corresponding activity and growth data indicate that Fe, S and Co were assimilated.

**FIGURE 3:**
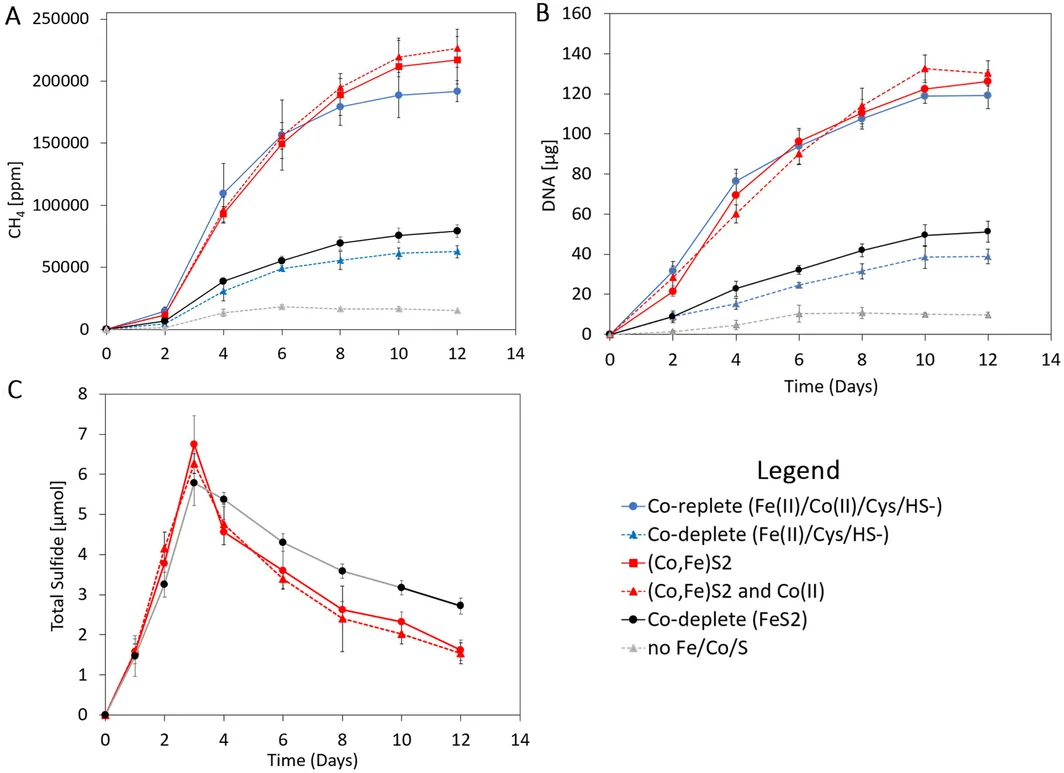
Production of methane (CH_4_, A), DNA as a proxy for biomass (B) and sulphide (C) in cultures of *Methanosarcina barkeri* Fusaro provided with acetate and methanol as methanogenesis substrates. Cultures were provided with synthetic [(Co,Fe)S_2_] nanoparticles or soluble sources of ferrous iron (Fe(II)), cobaltous cobalt Co(II) and sulphur (cysteine (Cys) and/or sulphide (HS^−^)) where indicated. Synthetic [(Co,Fe)S_2_] nanoparticles were added to a final concentration of 2 mM Fe. Fe(II), Co(II), Cys and HS^−^ were added to final concentrations of 20 μM, 2 μM, 2 mM and 50 μM, respectively, where indicated. Data shown are the mean and standard deviations of triplicate cultures. Production of HS^−^ was only measured in cultures grown with either FeS_2_ or [(Co,Fe)S_2_].

#### 3.3.3 M. maripaludis

Cultures of formate‐grown *M. maripaludis* were examined for their ability to use (Co,Fe)S_2_ as a source of Fe, S and Co, with similar controls to those mentioned above for *M. barkeri* . Cultures of *M. maripaludis* produced minimal CH_4_ and DNA when no Fe, S or Co were provided whereas cultures provided with soluble Fe, S and Co produced significant CH_4_ and DNA (Figure 4A,B). Cultures provided with soluble Fe and S but no Co or with FeS_2_ but no Co produced similar amounts of CH_4_ and DNA but less than positive controls, indicative of Co limitation. Interestingly, cultures provided with (Co,Fe)S_2_ produced CH_4_ and DNA at levels that were like the positive control. Cells grown with either FeS_2_ or (Co,Fe)S_2_ produced HS^−^ (Figure 4C) at levels consistent with prior characterization of the organism (Spietz, Payne, Kulkarni, et al. 2022; Payne et al. 2023). Unlike the *M. barkeri* cultures, amendment of (Co,Fe)S_2_‐grown *M. maripaludis* with soluble Co decreased the rate and extent of HS^−^ production as well as production of CH_4_ and DNA, possibly indicative of Co toxicity.

**FIGURE 4:**
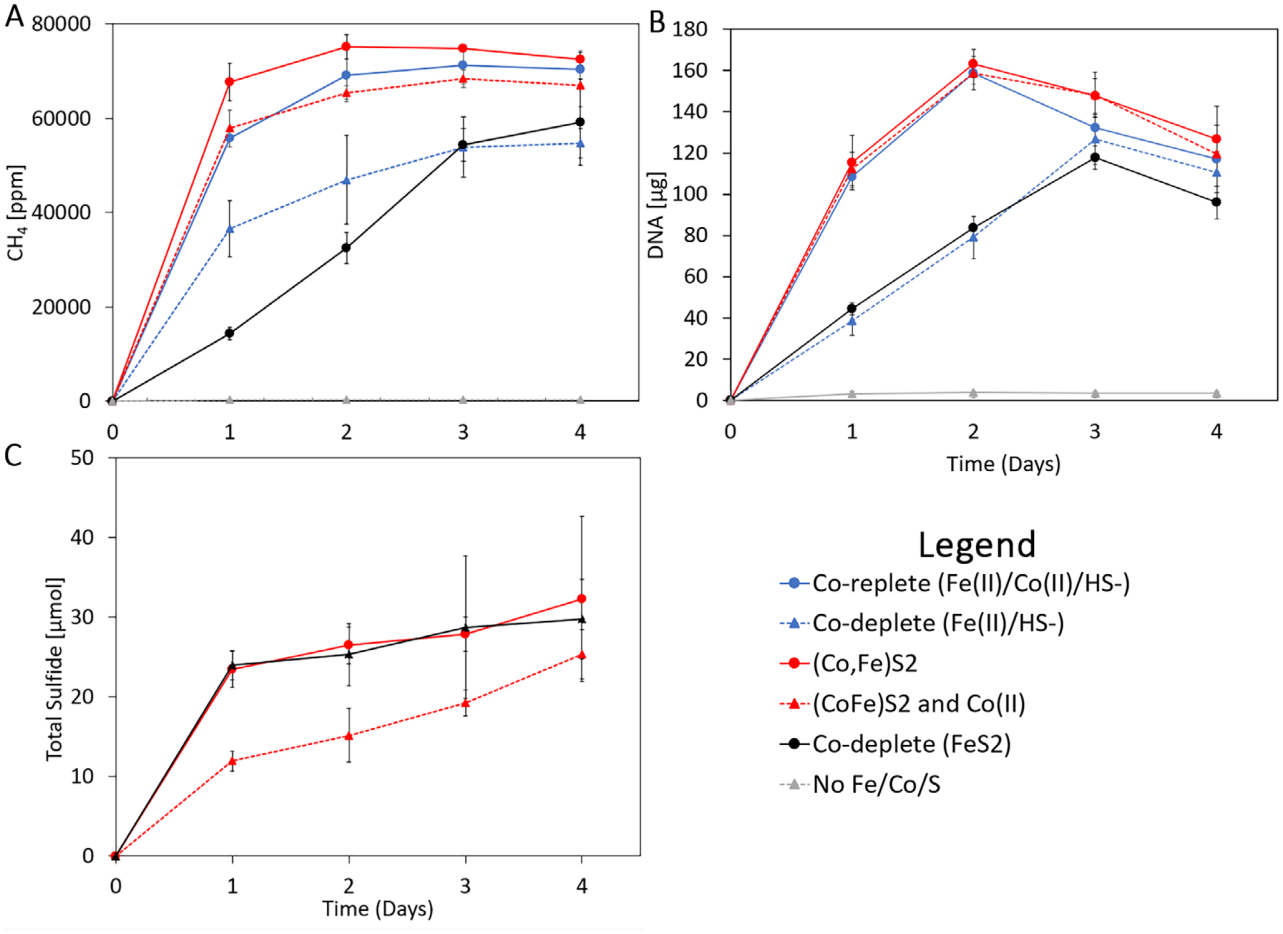
Production of methane (CH_4_, A), DNA as a proxy for biomass (B) and sulphide (HS^−^, C) in cultures of *Methanococcus maripaludis* S2 grown with formate as methanogenesis substrate. Cultures were provided with synthetic [(Co, Fe)S_2_] nanoparticles or soluble sources of ferrous iron (Fe(II)), cobaltous cobalt (Co(II)) and sulphide (HS^−^) where indicated. Synthetic [(Co,Fe)S_2_] nanoparticles were added to a final concentration of 2 mM Fe where indicated. Fe(II), Co(II) and HS^−^ were added to final concentrations of 20 μM, 2 μM and 2 mM, respectively, where indicated. Data shown are the mean and standard deviations of triplicate cultures. Production of HS^−^ was only measured in cultures grown with either FeS_2_ or [(Co,Fe)S_2_].

FeS_2_ reduction by methanogens results in the release of HS^−^ and formation of pyrrhotite (Fe_1−x_S) on the mineral surface (Spietz, Payne, Kulkarni, et al. 2022). Fe_1‐x_S is soluble with respect to Fe(II) but not S^2−^ (Mikhlin 2000), resulting in free Fe(II) in solution that can react with HS^−^ to form FeS_aq_ clusters (Spietz, Payne, Kulkarni, et al. 2022). At pH 7.0, these clusters remain small (< 20 nM in diameter) for periods of several hours, before precipitation as mackinawite (FeS_mack_) (Kour et al. 2025). Further, FeS_mack_ is relatively soluble (K_sp_ = 10^–3.5^; (Rickard and Luther 2007)) and re‐solubilization of this metastable mineral under low HS^−^ concentrations as FeS_aq_ functions to maintain a steady supply of FeS_aq_ in solution (Kour et al. 2025). As mentioned above, FeS_aq_ are thought to passively diffuse across the cell membrane and they are then trafficked to meet Fe and S biosynthetic demands (Payne, Shepard, et al. 2021). These collective phenomena may also help to explain the apparent utilization of Co from (Co,Fe)S_2_. Like cultures grown with FeS_2_ as sole Fe and S source, cultures grown with (Co,Fe)S_2_ as sole Co, Fe and S source produced HS^−^, indicating mineral reduction.

### 3.4 Requirement for Association With (Co,Fe)S_2_ During Growth

Methanogens directly associate with FeS_2_ and (Ni,Fe)S_2_ to facilitate reductive dissolution of the minerals (Payne, Spietz, and Boyd 2021; Spietz et al. 2023). FE‐SEM analysis of *M. maripaludis* grown with (Co,Fe)S_2_ revealed similar trends. Cells provided with soluble Fe, Co and S were ~700 nm in diameter (Figure S4A), whereas those grown with (Co,Fe)S_2_ were ~500 nm in diameter (Figure S4B). A similar decrease in cell diameter was measured in cultures of *M. voltae* when grown on FeS_2_ versus soluble forms of Fe and S and may be related to nutrient limitation, as previously suggested (Payne, Shepard, et al. 2021). Despite the decreased size, *M. maripaludis* cells grown with (Co,Fe)S_2_ were smooth, spherical and formed aggregates along the mineral surface. To investigate whether direct contact is required for the reductive dissolution of the mineral, we examined whether physical separation from the mineral was required for growth and activity.

Dialysis membranes with nominal pore sizes of 12–14 kDa were used to limit physical access of cells to (Co,Fe)S_2_ and to limit interactions of diffusible extracellular electron carriers with the mineral to less than this pore size. Importantly, some dialysis membranes interact with electron carriers (e.g., anthraquinone‐2,6‐disulphonate (AQDS)) and can prevent their passage despite having sufficiently large pore sizes (Nevin and Lovley 2000). However, our prior studies of FeS_2_ reduction have shown that carriers such as AQDS can pass through the membranes used in this study and that they can support reductive dissolution of FeS_2_ (Spietz et al. 2023). Controls included no Fe, S or Co amendment (negative control), amendment with soluble Fe, S and Co (positive control), as well as controls to examine whether activity/growth defects could be attributed to active mineral reduction versus abiotic mineral dissolution.

#### 3.4.1 M. barkeri

Methanol and acetate‐grown *M. barkeri* cultures readily grew when allowed direct contact with (Co,Fe)S_2_ as the sole source of Fe, Co and S (Figure 5A,C). Sequestration of the mineral in dialysis membranes with a 12–14 kDa pore size prevented growth, consistent with prior studies of *M. barkeri* that found direct contact was necessary for FeS_2_ and (Ni,Fe)S_2_ reductive dissolution (Spietz, Payne, Kulkarni, et al. 2022; Payne, Spietz, and Boyd 2021). However, when soluble Fe(II)/Cys/HS^−^ were added to sequestered (Co,Fe)S_2_, growth was observed. This suggests that the amount of Co abiotically solubilized (~20 nM, see above) from (Co,Fe)S_2_ or as reagent contaminant is sufficient to meet the Co demands of Fe(II)/Cys/HS^−^‐grown cells. It is important to note that a 75% reduction in DNA and CH_4_ production was observed in cultures grown with Fe(II)/Cys/HS^−^ without Co versus with Co (Co‐deplete versus Co‐replete conditions) whereas no reduction was observed in cultures grown with Fe(II)/Co(II)/Cys/HS^−^ versus those grown with Fe(II)/Cys/HS^−^ and Co as sequestered (Co,Fe)S_2_. This may indicate that *M. barkeri* requires lower amounts (~20 nM) of Co(II) than what is provided, which would potentially be consistent with the calculated amount of Co(II) remaining in reactors (> 84%) following harvesting of late log‐phase cells and accounting for Co in biomass in cultures grown with 2 μM Co(II). Much of the energy metabolism of *M. barkeri* is dependent on membrane‐associated redox active [Fe‐S] cluster‐ or cytochrome‐containing enzymes and lipid soluble methanophenazine‐like electron carriers (Mand and Metcalf 2019; Kulkarni et al. 2018). These enzymes and electron carriers perhaps provide suitable mechanisms for transporting electrons from the cytoplasm extracellularly to the mineral, either directly or via extracellular shuttles, as has been demonstrated here and previously (Spietz, Payne, Kulkarni, et al. 2022; Yee et al. 2019; Rowe et al. 2019). However, thorough mechanistic studies of this phenomenon still need to be conducted.

**FIGURE 5:**
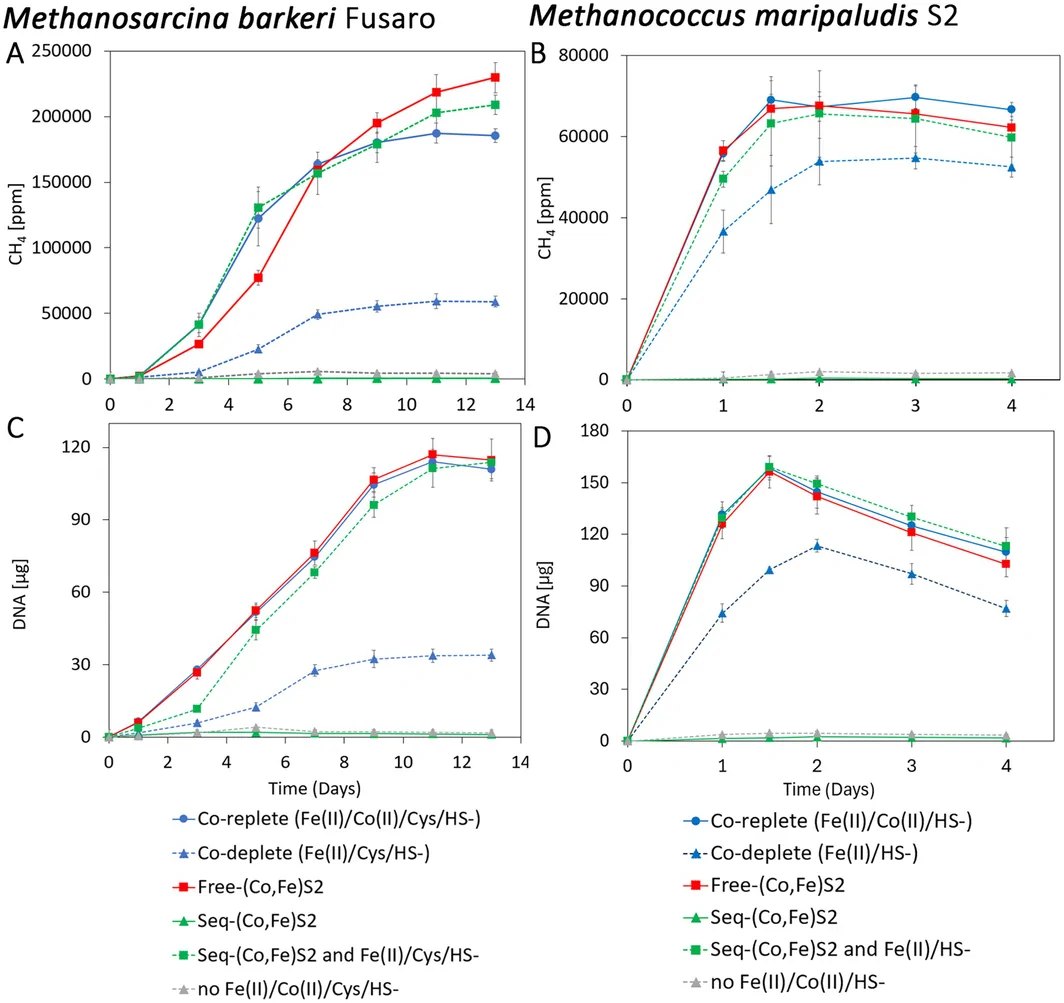
Production of methane (CH_4_, A, B) and DNA as a proxy for biomass (C, D) in cultures of *Methanosarcina barkeri* Fusaro (A, C) and *Methanococcus maripaludis* S2 (B, D) provided with direct or restricted access to synthetic colbaltoan pyrite [(Co,Fe)S_2_] nanoparticles. Cultures were provided with acetate and methanol ( *M. barkeri* ) or formate ( *M. maripaludis* ) as methanogenesis substrates. Cultures were provided with synthetic [(Co,Fe)S_2_] or ferrous iron (Fe(II)), cobaltous cobalt (Co(II)) and cysteine (Cys) and/or sulphide (HS^−^). Fe(II) Co(II), Cys, and/or HS^−^ were added to final concentrations of 20 μM, 2 μM, 2 mM and 50 μM ( *M. barkeri* ) or 2 mM ( *M. maripaludis* ), respectively, where indicated. (Co,Fe)S_2_ was added to a final concentration of 2 mM Fe and was provided free in solution (Free) or as sequestered in dialysis membranes with 12–14 kDa pore sizes (Seq), as indicated. Data shown are the mean and standard deviation of triplicate cultures.

#### 3.4.2 M. maripaludis

Formate‐grown *M. maripaludis* cultures were able to meet their Fe, Co and S requirements from (Co,Fe)S_2_ when allowed direct access to the mineral (Figure 5B,D). However, when the mineral was sequestered in dialysis membranes with a 12–14 kDa pore size, the amount of CH_4_ and DNA production was similar to that of the no Co, Fe and S negative control, consistent with other Methanococcales strains, for example, *Methanococcus voltae* , grown on FeS_2_ (Payne, Shepard, et al. 2021). While this suggests direct access is necessary for the reductive dissolution of the mineral, supplying soluble Fe(II) and HS^−^ rescued growth. Like *M. barkeri* , this indicates that the amount of Co(II) solubilized from the mineral abiotically (~20 nM, see above) is sufficient to meet the Co demands of Fe(II)/HS^−^‐grown *M. maripaludis* cells. Further, a 40% reduction in DNA and CH_4_ production was observed in cultures grown with Fe(II)/HS^−^ without Co versus with Co (Co‐deplete vs. Co‐replete conditions) whereas no reduction was observed in cultures grown with Fe(II)/Co(II)/HS^−^ versus those grown with Fe(II)/HS^−^ and with Co as sequestered (Co,Fe)S_2_. Thus, like *M. barkeri* , this may indicate that *M. maripaludis* requires lower amounts (~20 nM) of Co(II) than what is provided. This would also potentially be consistent with the calculated amount of Co(II) remaining in reactors (> 93%) following harvesting of late log‐phase cells and accounting for Co in biomass in cultures grown with 2 μM Co(II). Importantly, given that Methanococcales represent one of the earliest diverging lineages of methanogens (Wolfe and Fournier 2018), these results suggest that they evolved highly efficient mechanisms of Co acquisition early in their evolution and have maintained these capabilities to modern day where sub‐nM concentrations of soluble Co are common (Zerkle et al. 2005; Dupont et al. 2010; Anbar and Knoll 2002; Anbar 2008). Despite evidence the members of the Methanococcales can perform EET (Payne, Spietz, and Boyd 2021; Payne, Shepard, et al. 2021; Payne et al. 2023, 2024), membrane‐associated redox active enzymes or electron carriers in organisms in this lineage are far more restricted than *M. barkeri* (and other Class II/III methanogens) and include [NiFe]‐hydrogenases (Ehb/Eha) and Mtr but not lipid soluble electron carriers (e.g., quinones, methanophenozine) or cytochromes. Additional work is required to determine how this more ancient group of methanogens (Johnson et al. 2021) transfers electrons extracellularly, with implications ranging from reduction of FeS_2_ as demonstrated herein to other insoluble minerals such as iron oxides (Vargas et al. 1998) or S^0^ (Stetter and Gaag 1983).

### 3.5 Cobamide Synthesis From (Co,Fe)S_2_

The above studies provide indirect evidence that methanogens can use Co derived from (Co,Fe)S_2_ to produce biomass via CODH and CH_4_ via Mtr, Mta and CODH, all of which are cobamide‐dependent. *M. barkeri* is well known for its production of 5‐hydroxybenzimidazolylcobamide, especially when grown methylotrophically (Mazumder et al. 1987; Silveira et al. 1991). Thus, as a secondary method, we sought to detect and quantify production of 5‐hydroxybenzimidazolylcobamide in *M. barkeri* cells grown with methanol and acetate and with soluble or mineral sources of Co. Cultures were provided with either 2 μM of Co(II), 33.4 μM Co as (Co,Fe)S_2_ (if fully solubilized) or were grown under Co‐deplete conditions. The amount of 5‐hydroxybenzimidazolylcobamide (normalized to DNA yield) was nearly 7‐fold higher in cultures provided with Co than those not provided with Co (Figure 6). Cultures grown with (Co,Fe)S_2_ as the sole source of Co, Fe and S produced the same amount of 5‐hydroxybenzimidazolylcobamide as cultures grown with soluble Fe(II)/Co(II)/Cys/HS^−^. This indicates that Co from (Co,Fe)S_2_ is being assimilated by *M. barkeri* and trafficked for use in cofactor biosynthesis. Thus, it is clear that *M. barkeri* and other methanogens can access Co from (Co,Fe)S_2_ and facilitate the transformation of this metal into biologically relevant cofactors and coenzymes.

**FIGURE 6:**
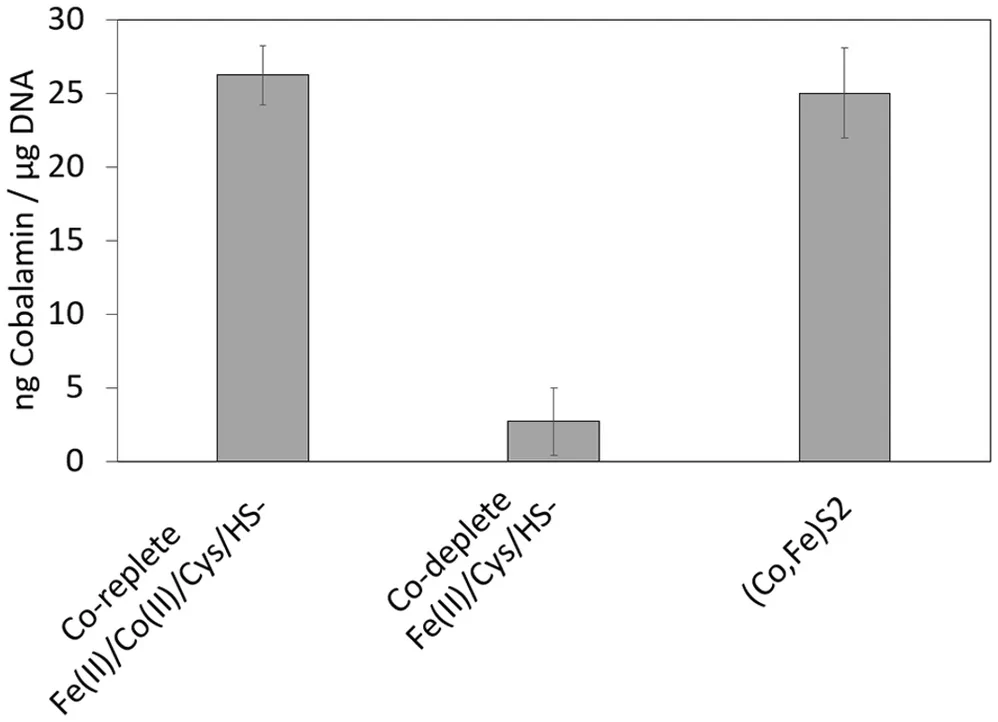
Production of cobalamides in cultures of *Methanosarcina barkeri* Fusaro provided with methanol and acetate as methanogenesis substrates. Cultures were grown with synthetic cobaltoan pyrite [(Co,Fe)S_2_] nanoparticles or with ferrous iron (Fe(II)), cysteine (Cys) and sulphide (HS^−^) as Fe and S sources in the presence and absence of soluble cobaltous Co (Co(II)), where indicated. (Co,Fe)S_2_ was added to a final concentration of 2 mM Fe. Fe(II), Cys, HS^−^ and Co(II) were added to final concentrations of 20 μM, 2 mM, 50 μM and 2 μM, respectively, where indicated. Data shown are the mean and standard deviation of triplicate cultures.

## Author Contributions

**Hunter Dulay:** formal analysis, data curation, investigation, methodology, validation, visualization, writing – original draft, writing – review and editing. **Md Gahangir Alam:** investigation. **Devon Payne:** writing – review and editing, conceptualization. **James Larson:** data curation, investigation, resources, writing – review and editing. **Brian Bothner:** resources, writing – review and editing, supervision. **Eric S. Boyd:** conceptualization, methodology, data curation, supervision, visualization, funding acquisition, project administration, writing – original draft, writing – review and editing, resources, formal analysis.

## Funding

This work was supported by the U.S. Department of Energy (DE‐SC0020246).

## Conflicts of Interest

The authors declare no conflicts of interest.

## Supporting information


**Figure S1:** Comparison of methods for monitoring growth and activity of *Methanosarcina barkeri* Fusaro and *Methanococcus maripaludis* S2 cultures. Production of cells (panels A, D), methane (CH_4_; panels B, E) and DNA (panels C, F). *M. barkeri* was grown with methanol and acetate as methanogenesis substrates with ferrous iron and sulphide as iron and sulphur source, respectively. *M. maripaludis* was grown with formate as the methanogenesis substrate and ferrous iron and cysteine/thiosulphate as iron and sulphur source, respectively. The data shown are the mean and standard deviation of triplicate cultures.
**Figure S2:** X‐ray diffraction spectra of synthetic pyrite (FeS_2_; black) and FeS_2_ containing 2.24% Co [(Co,Fe)S_2_; blue; see Table 2] (A). The best‐fit model spectrum for FeS_2_ (R/E = 1.38) and (Co,Fe)S_2_ (R/E = 2.32) are overlayed in purple. Peaks attributed to phases other than FeS_2_ are indicated for cobalt oxide (Co_3_O_4_; yellow), possible cobaltkieserite (Co(SO_4_)(H_2_O); red), possible cobaltpentlandite (Co_9_S_8_; orange), goethite (FeO(OH); green) and elemental sulphur (S^0^; dark blue). Phase weight‐percent values based on semi‐quantitative mineral analysis of the spectrum of (Co,Fe)S2 (B).
**Figure S3:** Field emission microscopy (FEM) image of synthetic cobaltoan pyrite [(Co,Fe)S_2_] (A). Energy‐dispersive X‐ray spectroscopy images indicating the co‐localized spatial distribution of sulphur (B), iron (C) and cobalt (D). The scale bar in each image is 50 μm.
**Figure S4:** Scanning electron micrographs of *Methanococcus maripaludis* S2 grown with formate and either (A) soluble Fe(II) Co(II), cysteine and thiosulphate or (B) synthetic (Co,Fe)S_2_ nanoparticles. Arrows in (B) highlight the coccoid cells among the mineral rhombohedral nanoparticles. Scale bars are 200 nm.

## Data Availability

The data that support the findings of this study are available on request from the corresponding author. The data are not publicly available due to privacy or ethical restrictions.
