# Supplementary material for: Influence of Soluble and Mineral Sources of Cobalt on Methanogenesis and Growth in Methanosarcina barkeri Fusaro and Methanococcus maripaludis S2
Source: Environ Microbiol. 2026 Nov 10;28(11):e70482. doi: 10.1111/1462-2920.70482 (PMC13749417; doi:10.1111/1462-2920.70482)
Supplement: Supplementary file 1 [file EMI-28-e70482-s001.docx]

**Supplemental Figure 1.** Comparison of methods for monitoring growth and activity of Methanosarcina barkeri Fusaro and Methanococcus maripaludis S2 cultures. Production of cells (panels **A, D**), methane (CH_4_; panels **B, E**), and DNA (panels **C, F**)**.** M. barkeri was grown with methanol and acetate as methanogenesis substrates with ferrous iron and sulfide as iron and sulfur source, respectively. M. maripaludis was grown with formate as the methanogenesis substrate and ferrous iron and cysteine/thiosulfate as iron and sulfur source, respectively. The data shown are the mean and standard deviation of triplicate cultures.

**Supplemental Figure 2.** X-ray diffraction spectra of synthetic pyrite (FeS_2_; black) and FeS_2_ containing 2.24% Co [(Co,Fe)S_2_; blue; *see* **Table 2**] (**A**). The best-fit model spectrum for FeS_2_ (R/E = 1.38) and (Co,Fe)S_2_ (R/E = 2.32) are overlayed in purple. Peaks attributed to phases other than FeS_2_ are indicated for cobalt oxide (Co_3_O_4_; yellow), possible cobaltkieserite (Co(SO_4_)(H_2_O); red), possible cobaltpentlandite (Co_9_S_8_; orange), goethite (FeO(OH); green), and elemental sulfur (S⁰; dark blue). Phase weight-percent values based on semi-quantitative mineral analysis of the spectrum of (Co,Fe)S2 (**B**).

**Supplemental Figure 3.** Field emission microscopy (FEM) image of synthetic cobaltoan pyrite [(Co,Fe)S_2_] (**A**). Energy-dispersive X-ray spectroscopy images indicating the co-localized spatial distribution of sulfur (**B**), iron (**C**), and cobalt (**D**). The scale bar in each image is 50 µm.

**Supplemental Figure 4.** Scanning electron micrographs of Methanococcus maripaludis S2 grown with formate and either (**A**) soluble Fe(II), Co(II), cysteine, and thiosulfate or (**B**) synthetic (Co,Fe)S_2_ nanoparticles. Arrows in (**B**) highlight the coccoid cells among the mineral rhombohedral nanoparticles. Scale bars are 200 nm.
